# Comparison between optical microscopy and the Sysmex XN‐3000 for schistocyte determination in patients suspected of having schistocytosis

**DOI:** 10.1002/hsr2.138

**Published:** 2019-11-29

**Authors:** Chattree Hantaweepant, Natthaporn Sasijareonrat, Boonyanuch Chutvanichkul, Khemajira Karaketklang, Yingyong Chinthammitr

**Affiliations:** ^1^ Division of Haematology, Department of Medicine, Faculty of Medicine Siriraj Hospital Mahidol University Bangkok Thailand; ^2^ Department of Medicine, Faculty of Medicine Siriraj Hospital Mahidol University Bangkok Thailand

**Keywords:** automated device, fragmented red cells, microscopic method, schistocytes, Sysmex XN

## Abstract

**Background and aims:**

Diagnosis of thrombotic microangiopathy (TMA) relies on microscopic schistocyte determination by an experienced microscopist. In addition, schistocytes can be found in non‐TMA–related disorders such as thalassaemia. We aimed to compare the accuracy of the automated haematology analyser Sysmex XN‐3000 for schistocyte detection, to that of the microscopy approach, in patients suspected of having schistocytosis.

**Methods:**

Consecutive blood samples were collected between April 2016 and March 2017 at Siriraj Hospital, Mahidol University, Bangkok, Thailand. Specimens were collected from adults with suspected TMA or with thalassaemia trait and/or disease. All blood samples were examined by both microscopy and the analyser. Samples were considered to be positive for schistocytes (ie, schistocytosis) if they had a schistocyte count ≥1% by microscopy. The analyser's ability to determine schistocytosis was assessed by receiver operating characteristic (ROC) curve. Sensitivity, specificity, positive (PPV), and negative predictive value (NPV) of an appropriate cut‐off point were calculated, with manual microscopy as the standard. Quantitative agreement in schistocyte counts between the two approaches was assessed using 95% limits of agreement, Bland‐Altman plots, intraclass correlation coefficient, and concordance correlation coefficient.

**Results:**

Ninety‐seven blood samples (62 suspected TMA and 35 thalassaemia) were collected. ROC curve analysis of the analyser for determining schistocytosis showed an area under the curve of 0.803 (95% confidence interval, 0.689‐0.917, *P* < 0.001). A cut‐off point of 0.6% yielded 86.1% sensitivity, 77.8% specificity, 94.4% PPV, and 56.0% NPV. The automated schistocyte count did not quantitatively agree with schistocyte counts by microscopy, neither in all blood specimens (mean of difference: −1.09; 95% limits of agreement, −11.9 to 9.7) nor in the subgroups (TMA, −0.88; 95% limits of agreement, −6.60 to 4.84; thalassaemia, −2.4; 95% limits of agreement, −14.10 to 9.30). The differences in the estimation of fragmented red blood cells between the methods tended to increase at higher schistocyte counts.

**Conclusion:**

Sysmex XN‐3000 can be used for qualitative measurement of schistocytosis, but should not be used as a quantitative tool for schistocyte counting. Improvements are needed before this analyser's schistocyte detection feature can be recommended for use in clinical practice.

## 1 INTRODUCTION

Schistocytes are fragmented red blood cells (FRCs) that arise from the mechanical destruction of red blood cells (RBCs). They indicate the possible presence of a thrombotic microangiopathy (TMA), such as thrombotic thrombocytopenic purpura (TTP), haemolytic uremic syndrome (HUS), and disseminated intravascular coagulation (DIC).^1^ Both TTP and HUS are life‐threatening conditions that require an urgent diagnosis and appropriate treatment.^2^, ^3^, ^4^ Therefore, schistocyte identification is one of the most important cornerstones of diagnosis for these two diseases.^2^, ^3^, ^4^ Presentation of schistocytes in TMA is a dominant feature in peripheral blood smears; however, schistocytes are not specific to TMA.^5^ Schistocytes can also be found in non‐TMA–related disorders and conditions such as thalassaemia and inherited red blood cell (RBC) membrane defects, and as a consequence of mechanical damage from prosthetic heart valves, structural abnormalities of great vessels, and malignant hypertension.^6^, ^7^, ^8^ Schistocytes identified in peripheral blood smears of non‐TMA–related diseases are usually just one of many RBC shape abnormalities.^5^

The gold standard for the identification of schistocytes is microscopic evaluation by well‐trained personnel.^5^, ^9^ In the past, automated machines could not accurately determine blood cells with abnormal morphology and, therefore, every flagged result from an automated device required confirmation by a clinician or technologist.^10^ However, this manual approach to schistocyte determination has limitations in routine clinical practice, including a lack of intra‐ and interdepartmental standardization, the fact that it is time‐consuming—as argued by Lesesve^6^—and the fact that confirmation needs to be made by a highly experienced physician.^4^, ^11^ In patients suspected of having TTP, a lack of availability of experienced personnel for microscopy scoring could cause delays in both diagnosis and treatment, which could lead to unfavourable outcomes.

For the present study, the automated Sysmex XN‐3000 (Sysmex Corporation, Kobe, Japan) was selected for comparison with microscopy because it includes a function for the detection of schistocytes, and it is already used at our centre for routine laboratory tests, including complete blood count, nucleated red blood cell count, and reticulocyte count. Furthermore, the Sysmex XN‐3000 can count an average of 30 000 RBCs simultaneously, whereas only about 1000 RBCs can be examined microscopically in one peripheral blood smear, and with reported poor reproducibility.^9^

In this study, we use the terms “schistocytosis” for schistocyte counts more than or equal to 1% by microscopy, “schistocyte” for microscopy evaluation, and “fragmented red blood cell (FRC)” for machine estimation. To date, no study has compared the FRC count obtained from the Sysmex XN series with the schistocyte count yielded by the well‐established microscopy method. The current study aimed to investigate the concordance between the Sysmex XN‐3000 and the manual counting of schistocytes in a heterogeneous group of patients, which included those with TMA or thalassaemia. We hypothesized that the Sysmex XN‐3000 device might be an appropriate tool for accurately determining schistocyte count. If so, this would obviate the need for highly trained and experienced personnel to confirm the presence of schistocytes and could reduce the time to diagnosis and treatment.

## 2 MATERIALS AND METHODS

### 2.1 Blood specimens

All peripheral venous blood samples were collected between 1 April 2016 and 31 March 2017 at Siriraj Hospital, Mahidol University, Bangkok, Thailand. The specimens, which were collected consecutively from patients older than 17 years of age, were obtained on the basis of a suspected diagnosis of TMA (TTP, DIC, malignant hypertension, and systemic lupus erythematosus) by the treating physician, as well as for known cases of thalassaemia trait and disease. We aimed to investigate schistocytes in blood samples from patients with suspected TMA because we wanted to evaluate the diagnostic accuracy of the automated machine for this specific group of patients. However, diseases with TMA, such as TTP^12^ and HUS,^13^ are rare. Therefore, we also included blood samples from patients with thalassaemia trait and disease due to the ease of detecting schistocytes in peripheral blood smears. The enrolment criteria for thalassaemia were patients with non‐transfusion–dependent thalassaemia, with or without a splenectomy, who had received their last blood transfusion more than 3 months beforehand. The reason that we chose this type of thalassaemia was because there would be no dilutional effect from a blood transfusion to interfere with the evaluation of the schistocyte percentage. We collected the FRC count by the Sysmex XN‐3000, the schistocyte percentage by microscopy from three observers, haemoglobin (Hb) levels, white blood cell (WBC) count, platelet (PLT) count (these three parameters from the Sysmex XN‐3000), final diagnosis of patients with suspected TMA (from discharge summary notes by the treating physician), and diagnosis of thalassaemia trait and disease (from the hospital's medical records). A pilot series of 20 consecutive samples was used to evaluate the level of agreement among three observers. If the consistency between the evaluators was high, one observer would then participate in the comparison with the device (see below).

### 2.2 Automated method

FRCs were tested in the Sysmex XN‐3000 using ethylenediaminetetraacetic acid blood in a reticulocyte channel, according to the manufacturer's protocol. Therefore, the FRC parameter was presented only when requesting a blood test for the reticulocyte mode. A 3‐mL aliquot of each sample was measured within 3 hours of blood sampling to prevent spuriously high FRC percentage following delay of analysis. A study by Banno et al showed that the FRC percentage increased with time, reaching 14% 24 hours after blood sampling.^14^

The Sysmex XN‐3000 identifies FRCs by fluorescence‐based flow cytometry.^15^ This feature uses fluorochrome to stain ribosomal RNA and forward light scattering to detect blood cell size. Therefore, events which have less ribosomal RNA content than PLT and that are smaller than mature RBCs, are counted as FRCs.^15^, ^16^ About 30 000 RBCs can be analysed simultaneously, and the FRCs are automatically presented as a percentage appearing below the RBC area.^9^, ^15^ The Sysmex XN‐3000 used in this study was validated daily by internal quality control procedures, consistent with the manufacturer's instructions. There was also a monthly external quality control assessment by Randox International Quality Assessment Scheme from Randox Laboratories, United Kingdom.

### 2.3 Microscopy method

Consistent with the recommendations of the Schistocyte Working Group of the International Council for Standardization in Haematology (ICSH),^5^, ^17^ schistocytes were counted microscopically among 1000 RBCs (five oil fields) at 1000x magnification in optimal areas (where RBCs were appropriately separated from each other) of a peripheral blood smear, with May‐Grünwald‐Giemsa staining. The Sysmex XN‐3000 has slide‐making and slide‐staining functions, so 600 μL of blood was used to make one peripheral blood smear according to the manufacturer's protocol. The schistocyte numbers were then calculated as a percentage of RBCs. Only triangular schistocytes, helmet cells, and keratocytes were considered to be schistocytes. A positive cut‐off value of 1% schistocytes or higher was considered to be evidence of schistocytosis.^5^

The aforementioned three observers were a haematologist, a haematology resident, and a technician. Before the microscopy measurements were performed, these three observers were instructed about the morphological definition of schistocytes in the ICSH recommendations. They then examined and determined schistocytes on peripheral blood smears of known samples of schistocytosis (that were not included in the study) together, until everyone was confident that they had a complete and similar understanding of the criteria (see additional observer‐related details in the Supplementary information). Blinded to the diagnosis, the observers then independently assessed 20 blood specimens, which were a subset of the whole sample set, in order to calculate the level of agreement between two of them (intraclass correlation coefficient: ICC). An ICC greater than 0.75 indicates strong agreement between observers,^18^ and this would indicate that for the purposes of this study, any of the observers could determine the schistocyte percentage in the remaining peripheral blood smears.

### 2.4 Interfering factors

We additionally attempted to identify factors that may be associated with potential method disagreements. Hb, WBC, and PLT were selected on the basis of the fact that they might interfere with FRC quantification. Hb was selected because the common cause of anaemia is microcytic anaemia (eg, iron deficiency anaemia and thalassaemia).^19^, ^20^ Some RBCs with extremely small size can be counted as schistocytes,^16^, ^21^, ^22^, ^23^ which results in a falsely high FRC count. For WBC, some conditions that cause leukocytosis (eg, sepsis and acute leukaemia) can produce cytoplasmic fragments of WBC.^23^, ^24^ The small particles can be counted as schistocytes and lead to a falsely elevated FRC number.^22^ Regarding PLT, small schistocytes with a volume as low as that of PLT can be falsely identified as PLT.^23^ Therefore, conditions that have numerous small schistocytes might result in a spuriously high PLT count.^23^, ^25^

### 2.5 Ethical considerations

This non‐interventional study was approved by the Siriraj Institutional Review Board (SIRB) (Certificate of Approval no. Si805/2016). The committee waived the need to obtain consent for the collection, analysis, and publication of the anonymized data; for the production of peripheral blood smears; and, for the testing of the FRC count, given that these were left‐over blood samples obtained during routine clinical care.

### 2.6 Statistical analysis

The sample size was calculated using schistocyte detection data from a previous study^21^ (accuracy rate of 0.73 with an error of 0.09). Using a significance level of 0.05, a total of 94 samples was calculated. For the pilot samples, the ICC estimates, and their 95% confidence intervals (CIs) on the basis of a single rater, absolute agreement, and the 2‐way random‐effects model were used to assess the degree of agreement between two observers.^26^ Twenty blood samples for a pilot series were estimated on the basis of the assumption that an agreement between observers already exists (ICC = 0.7 in the null hypothesis), and that a higher degree of agreement between them is expected (ICC = 0.9 in the alternative hypothesis).^27^

For the assessment of the analyser's accuracy, a receiver operating characteristic (ROC) curve was used to evaluate the analyser's ability to detect a microscopically determined schistocyte count of greater than or equal to 1%. The area under the curve (AUC) of the analyser, and the sensitivity, specificity, positive predictive value (PPV), and negative predictive value (NPV) of different cut‐off points were calculated (see interpretation of AUC in the Supplementary information). Ninety‐five percent limits of agreement, Bland‐Altman plots, ICC, and concordance correlation coefficients were used to evaluate quantitative agreement between the automated device and the microscopy method for the complete sample set of samples and by subgroup (TMA and thalassemia). Scatter plots were used to evaluate the relationship between two variables. Because of the non‐normal distribution of variables, Spearman's rank correlation coefficient (*r*_s_) was used to evaluate correlation between the difference in schistocyte percentage (analyser‐microscopy) and other laboratory results (Hb, WBC, and PLT) (see interpretation of *r*_s_ in the Supplementary information). Considering their non‐normal distribution, schistocyte counts by microscopy for each disease are shown as median, minimum, and maximum. All statistical analyses were performed using SPSS Statistics for Windows, version 18 (SPSS Inc, Chicago, Illinois). A two‐sided *P* value of 0.05 or less was considered statistically significant.

## 3 RESULTS

### 3.1 Patient population, final diagnosis, and schistocyte percentage for each disease by the microscopy method

Ninety‐seven blood specimens were evaluated, and the blood samples were divided into two groups—the TMA group and the thalassaemia group. The ratio of blood samples from patients in the TMA group to patients in the thalassaemia group was 2:1. The TMA group included 62 blood samples from patients with suspected TMA (the final diagnosis included DIC, TTP, TMA, malignant hypertension, systemic lupus erythematosus, myelodysplastic syndrome, anaemia of chronic disease, and chronic kidney disease). The thalassaemia group included 35 blood samples from the beta thalassaemia trait, homozygous haemoglobin E, and β‐thalassaemia/Haemoglobin E disease (with and without splenectomy) (Table 1).

**Table 1:** Schistocyte percentage per 1000 RBCs (at 1000 power magnification) by microscopy for each evaluated hematologic disease

Diagnosis	n	Median (min‐max)
**TMA group**	**62**	
DIC	16	3.3% (0.0%‐7.7%)
TTP	6	6.1% (3.3%‐10.3%)
TMA	2	4.8% (3.9%‐5.7%)
Malignant HT	2	5.2% (4.5%‐5.9%)
SLE	4	2.0% (1.4%‐3.2%)
Other	32	1.0% (0.0%‐14.3%)
**Thalassaemia group**	**35**	
BE without splenectomy	20	7.7% (1.6%‐15.8%)
BE with splenectomy	10	4.4% (2.3%‐14.1%)
B trait	4	2.6% (1.3%‐4.0%)
Homo E	1	2.1% (2.1%‐2.1%)
**Total**	**97**	**3.3% (0.0**%**‐15.8%)**

*Note.* Other: myelodysplastic syndrome, anaemia of chronic disease, and chronic kidney disease.

Abbreviations: BE, β‐thalassaemia/Haemoglobin E disease; B trait, Beta thalassaemia trait; DIC, disseminated intravascular coagulation; Homo E, Homozygous haemoglobin E; malignant HT, malignant hypertension; SLE, systemic lupus erythematosus; TMA, thrombotic microangiopathy; TTP, thrombotic thrombocytopenic purpura

We included blood samples of patients initially suspected of having schistocytosis. Therefore, the prevalence of peripheral blood smears with schistocytosis in our study was 81.4%. The remaining negative samples (less than 1% schistocytes by manual count) were from 16 patients with other diseases (ie, myelodysplastic syndrome, anaemia of chronic disease, and chronic kidney disease) and two cases with DIC. The median schistocyte count for all specimens was 3.3% (Table 1). The highest median schistocyte count of 7.7% was found in patients with β‐thalassaemia/haemoglobin E disease, followed by TTP at 6.1% and malignant hypertension at 5.2%. Patients with other diseases had the lowest median schistocyte count, at 1.0%.

### 3.2 Pilot determination of interobserver agreement

The pilot series of 20 blood samples included six cases with a final diagnosis of DIC, two cases of systemic lupus erythematosus, one case of TMA, one case of TTP, nine cases of other diseases (myelodysplastic syndrome, anaemia of chronic disease, and chronic kidney disease), and one case of beta thalassaemia trait. The ICC between the haematologist and the haematology resident was 0.983 (95% CI, 0.956‐0.993); the ICC between the haematology resident and the technician was 0.985 (95% CI, 0.963‐0.994); and the ICC between the haematologist and the technician was 0.962 (95% CI, 0.907‐0.985). Since an ICC greater than 0.75 indicates strong agreement between observers, we decided that the haematology resident would assess the remainder of the specimens while blinded to all clinical information.

### 3.3 Comparison between standard microscopy and automated analyser

**Figure 1:**
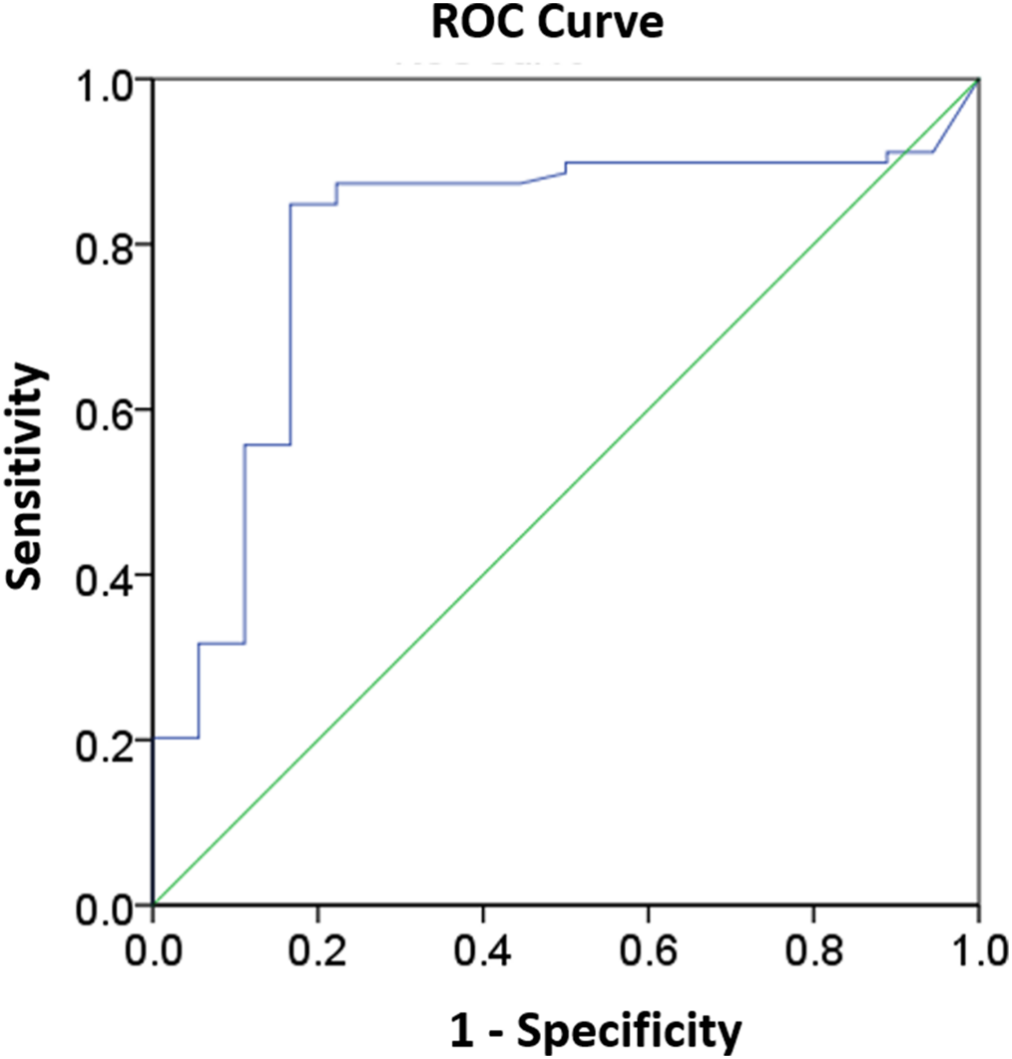
Receiver operating characteristic (ROC) curve to assess the accuracy of the Sysmex XN‐3000 for the discrimination between presence and absence of schistocytosis (schistocyte count greater than or equal to 1% by microscopy). The graph shows an area under the curve (AUC) of 0.803 (95% CI, 0.689‐0.917, *P* < 0.001)

We evaluated the accuracy of the analyser's ability to discriminate between presence and absence of schistocytosis (schistocytes greater than or equal to 1% by microscopy) by the ROC curve (Figure 1). The ROC curve analysis showed an AUC of 0.803 (95% CI, 0.689‐0.917, *P* < 0.001). At different cut‐off points, different sensitivity, specificity, PPV, and NPV were observed (Table 2).

**Table 2:** Sensitivity, specificity, positive predictive value (PPV), and negative predictive value (NPV) of different cut‐off points for detection of schistocytosis (schistocytes greater than or equal to 1% by microscopy) by the Sysmex XN‐3000, with the manual count as standard

Cut‐off point (%)	Sensitivity (%)	Specificity (%)	PPV (%)	NPV (%)
>0.4	87.3	61.1	90.8	52.4
>0.6	86.1	77.8	94.4	56
>0.8	84.8	77.8	94.4	53.8
>1.0	78.5	83.3	95.4	46.9
>2.0	55.7	83.3	93.6	30

**Figure 2:**
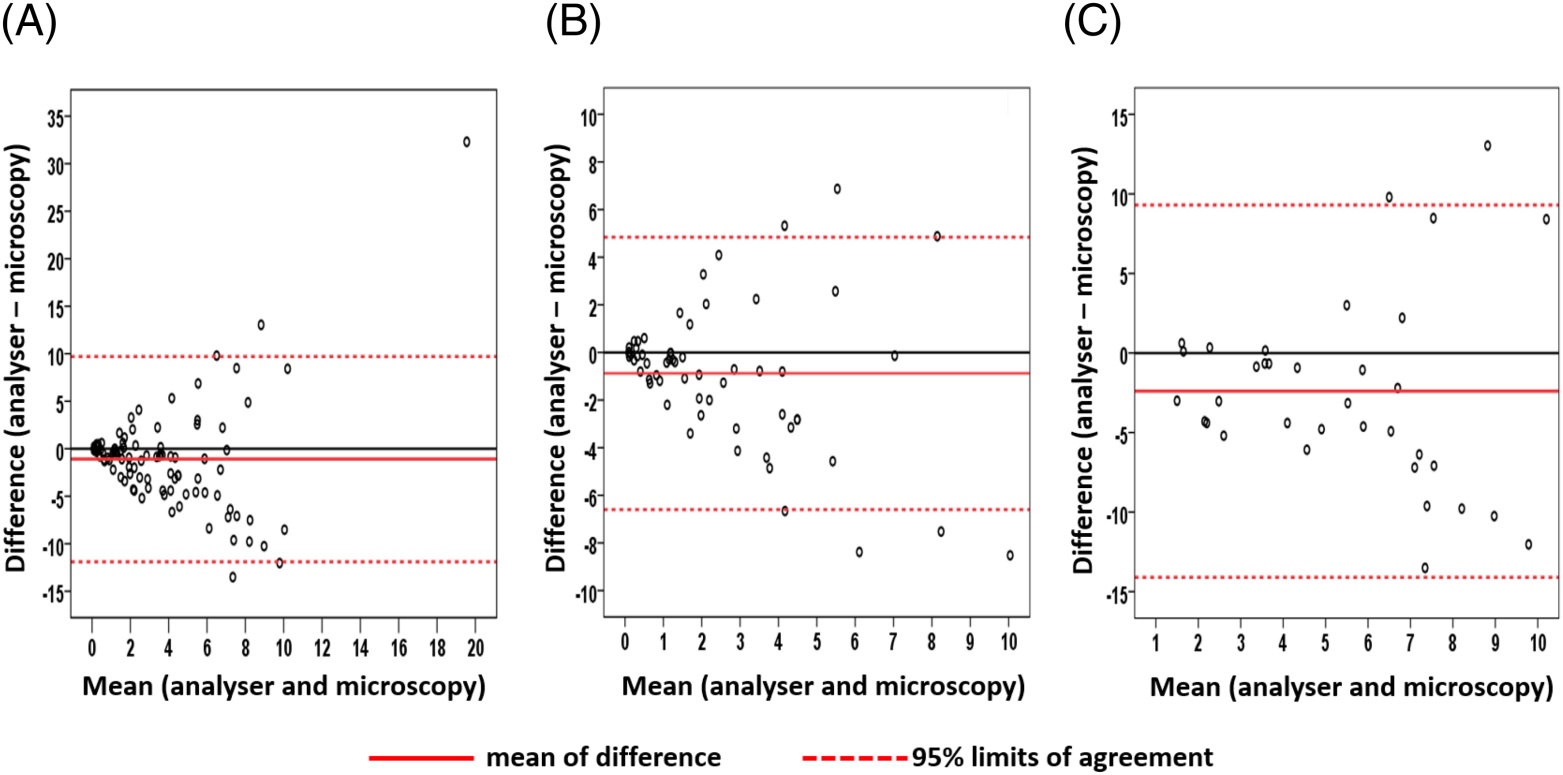
Bland‐Altman plots show disagreement in schistocyte percentage between the Sysmex XN‐3000 analyser and microscopy. A, For all blood specimens, the mean difference (analyser‐microscopy) was −1.09 (95% limits of agreement, −11.86 to 9.68). B, For patients with suspected TMA, the mean difference (analyser‐microscopy) was −0.88 (95% limits of agreement, −6.60 to 4.84). C, For the thalassaemia group, the mean difference (analyser‐microscopy) was −2.40 (95% limits of agreement, −14.10 to 9.30)

We determined the quantitative agreement between the two approaches in 97 samples. The analyser measured the FRC percentage at a mean of 1.09 units less than the observer (95% limits of agreement, −11.86 to 9.68) (Figure 2A). We next analysed samples by subgroup. For the group of patients with suspected TMA, the analyser measured FRC percentage a mean of 0.88 units less than the observer (95% limits of agreement, −6.60 to 4.84) (Figure 2B), while in the thalassaemia group, the analyser measured FRC percentage a mean of 2.40 units less than the observer (95% limits of agreement, −14.10 to 9.30) (Figure 2C). In all samples and in both subgroups, the difference in results between the two methods tended to increase as the mean schistocyte count of the two measurements increased.

### 3.4 Relationship between the disagreement in schistocyte percentage between the methods and potential interfering factors

**Figure 3:**
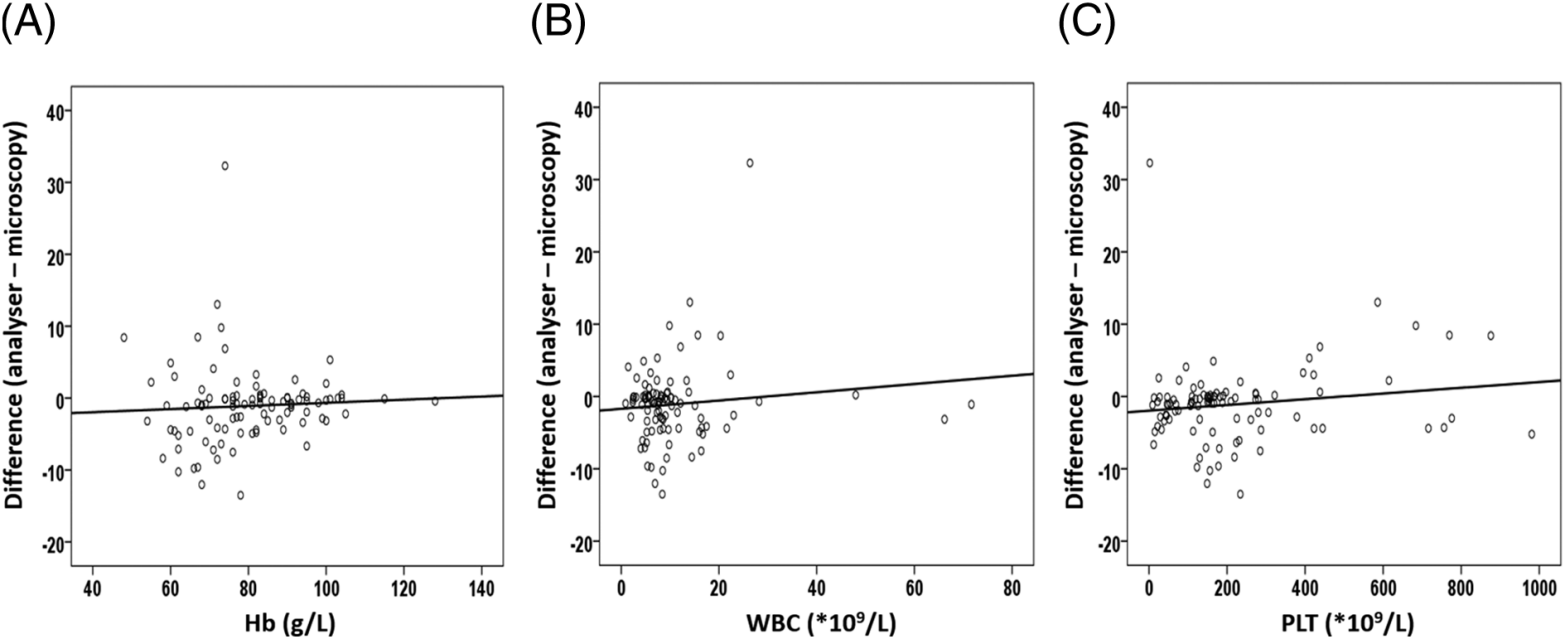
Relationship between the disagreement in schistocyte percentage between the Sysmex XN‐3000 and microscopy, and potential interfering factors, for the complete set of samples. A,Disagreement in schistocyte measurement poorly correlates with haemoglobin (Hb) (*r*_s_ = 0.187, 95% CI, −0.012 to 0.373, *P* = 0.07). B,Disagreement in schistocyte measurement poorly correlates with white blood cell (WBC) count (*r*_s_ = −0.030; 95% CI, −0.227 to 0.171, *P* = 0.77). C,Disagreement in schistocyte measurement poorly correlates with platelet (PLT) count (*r*_s_ = 0.128; 95% CI, −0.073 to 0.320, *P* = 0.21)

We sought to determine whether parameters determined by the Sysmex XN‐3000, including Hb levels, WBC count, and PLT count, could affect the difference in schistocyte detection between the two methods. The correlation between the disagreement in schistocyte measurements and each of the analyser's parameters that were assessed was poor (Figure 3). A previous study reported the FRC count to be falsely high in samples taken from patients with microcytic anaemia.^16^ We then analysed the correlation between the difference in schistocyte measurements and Hb specifically in the group of patients with thalassaemia trait and disease, because both conditions have microcytic RBCs.^28^ The correlation was also found to be poor (*r*_s_ = 0.051) (95% CI, −0.287 to 0.378, *P* = 0.77) (see Figure S1 in the Supplementary information).

## 4 DISCUSSION

This study is the first to compare schistocyte detection and quantification by the Sysmex XN series with that of the gold standard microscopy method in blood samples taken from patients suspected of having schistocytosis. Previous studies either researched FRC count by the Sysmex XE series (XE‐2100^1^, ^14^, ^16^, ^29^, ^30^ and XE‐5000^9^) in heterogeneous groups of patients, or studied the FRC reference range using an XN‐9000 in healthy patients.^15^ We found that when using the analyser in the samples derived from the group of patients suspected of having schistocytosis, it could discriminate between presence and absence of schistocytosis reasonably well, as evidenced by the AUC of the ROC curve being more than or equal to 0.8 (AUC = 0.803; 95% CI, 0.689‐0.917).^31^ However, given the 95% CI, the data is also compatible with AUC values less than or equal to 0.7, which suggests that the machine may possibly yield an insufficiently accurate result.^31^, ^32^ We then set forth to identify a cut‐off point with high sensitivity and an appropriately high specificity, because we specifically did not want to miss patients with TMA. Our analysis revealed a schistocyte count cut‐off point of greater than or equal to 0.6% to be optimal under our conditions, with a sensitivity of 86.1%, specificity of 77.8%, PPV of 94.4%, and NPV of 56%. However, because of the low NPV of this cut off, it requires microscopic examination to confirm the true negative result.

For quantitative measurement, our findings demonstrate that the Sysmex XN‐3000 does not agree with the results of microscopy, neither in the analysis including all blood samples nor by subgroup. Bland‐Altman plots showed substantial difference in schistocyte count (95% limits of agreement) between the two methods. FRC estimation by the automated method tends to be progressively different from the measurement by microscopy when the schistocyte count increases. From a clinical significance standpoint, these levels of disagreement are unacceptable. Underestimations of schistocyte percentage can result in false negatives for the presence of schistocytosis, which is critical for diagnosis of TMA. Furthermore, when the automated device overestimates FRC count, it cannot correctly monitor the levels of schistocytes after treatment.

The disagreement in quantitative assessment observed in our study does not support the findings of other studies that have reported a correlation between the FRC percentage obtained by the Sysmex XE‐2100 and manual counting. For example, Banno et al studied blood samples of five patients with schistocytosis in peripheral blood smears and reported a high correlation coefficient, of 0.952.^14^ Jiang et al collected blood samples from 100 patients with diseases such as DIC and HUS, and found a high correlation coefficient (Fisher *Z*‐transformation, *r* = 0.902).^16^ The results of these studies, however, cannot be directly compared with ours because they used correlation coefficient to test the agreement between the two tests, which is different from the statistical method used in our study. Chalvatzi et al reported, using a Bland Altman plot, that the Sysmex XE‐5000 overestimated the FRC percentage by a mean of 0.83 units (95% limits of agreement, −1.5 to 3.2) compared with that of microscopy, on 200 blood samples.^9^ The observed magnitude of disagreement of the Sysmex XE‐5000 was not as large as that observed for the Sysmex XN‐3000 in our study. One previous study reported that the principles of FRC detection have not changed from the XE‐series (2008) to the current XN‐series of analysers.^15^ However, the substantially quantitative disagreement of the Sysmex XN‐3000 in our study should stimulate more research to identify ways to improve the FRC detection method of the XN series.

Regarding the Hb level as an interfering factor, previous studies have reported conflicting findings. Jiang et al^16^ reported that the FRC count by the Sysmex XE‐2100 was spuriously high in samples taken from patients with iron deficiency anaemia. In contrast, previous studies using the Sysmex XE‐5000^9^ and XN‐9000^15^ devices have reported that an increased number of microcytic RBCs did not interfere with the FRC count, which is similar to the result we observed in our thalassaemia group. The reason for this difference between studies may be improvement in the function of subsequent generations of automated analysers. For WBC, our findings do not show a correlation between WBC count and the differences between the analyser and the microscopy analysis. This may be explained by the fact that we did not include patients with acute leukaemia, and that granulocytic fragments are not produced in all cases with leukocytosis from sepsis.^24^ Regarding PLT, our study shows that the PLT count does not appear to influence the disagreement of schistocyte measurements between the Sysmex XN‐3000 and microscopy. The present study is the first to evaluate WBC and PLT as interfering factors for FRC detection by an automated device.

Our study has some limitations. First, we did not include blood samples from normal individuals, which would be better to ascertain specificity of the analyser and establish a normal range of schistocyte counts as determined by microscopy; we used a negative cut‐off value of less than 1% schistocytes, per ICSH recommendations. Second, we did not collect mean corpuscular volume (MCV) in each sample to analyse the relationship with disagreement between the two tests. We assumed that thalassaemia trait and disease always have low MCV. Consequently, we cannot confidently conclude that the disagreement between the two measurements was not influenced by microcytic RBCs.

## 5 CONCLUSIONS

The FRC parameter from the Sysmex XN‐3000 can determine the qualitative presence of schistocytosis with reasonable accuracy, but microscopic examination is needed to confirm the absence of schistocytes when the machine reports a negative result. Importantly, however, our results strongly suggest that the XN‐3000 is not a good tool for quantitatively determining schistocyte counts. Further refinement of its automated schistocyte counting feature is required before the Sysmex XN‐3000 can be recommended for clinical evaluation.

## FUNDING

This study was supported by a research grant from the Thai Society of Haematology. Our funding source had no role in study design; collection, analysis, and interpretation of data; writing of the report; and the decision to submit the report for publication.

## CONFLICTS OF INTEREST

The authors have declared that there is no conflict of interest.

## AUTHOR CONTRIBUTIONS

Conceptualization: Yingyong Chinthammitr

Data Curation: Natthaporn Sasijareonrat

Formal Analysis: Khemajira Karaketklang, Chattree Hantaweepant

Investigation: Natthaporn Sasijareonrat, Boonyanuch Chutvanichkul, Chattree Hantaweepant

Writing‐Original Draft Preparation: Chattree Hantaweepant

Writing‐Review and Editing: Yingyong Chinthammitr, Natthaporn Sasijareonrat, Boonyanuch Chutvanichkul, Khemajira Karaketklang, Chattree Hantaweepant

All authors have read and approved the final version of the manuscript.

Chattree Hantaweepant had full access to all of the data in this study and takes complete responsibility for the integrity of the data and the accuracy of the data analysis.

## TRANSPARENCY STATEMENT

Chattree Hantaweepant affirms that this manuscript is an honest, accurate, and transparent account of the study being reported; that no important aspects of the study have been omitted; and, that any discrepancies from the study as planned (and, if relevant, registered) have been explained.

## Supporting information


**Figure S1.** Relationship between disagreement in schistocyte percentage between the analyser and microscopy, and haemoglobin (Hb) for the thalassaemia group. Disagreement in schistocyte measurement poorly correlates with Hb (*r*_s_ = 0.051, 95% CI: ‐0.287‐0.378, *P* = 0.77).

## Data Availability

The data that support the findings of this study are available from the corresponding author upon reasonable request.
