# Supplementary material for: Comparison between optical microscopy and the Sysmex XN‐3000 for schistocyte determination in patients suspected of having schistocytosis
Source: Health Sci Rep. 2019 Nov 29;3(1):e138. doi: 10.1002/hsr2.138 (PMC7060895; doi:10.1002/hsr2.138)
Supplement: Supplementary file 1 — Figure S1. Relationship between disagreement in schistocyte percentage between the analyser and microscopy, and haemoglobin (Hb) for the thalassaemia group. Disagreement in schistocyte measurement poorly correlates with Hb (r s = 0.051, 95% CI: ‐0.287‐0.378, P = 0.77). [file HSR2-3-e138-s001.docx]

**Supplementary Information**

1. **Supplementary Materials and Methods**

***Microscopy method***

The reasons why the three observers were instructed about the morphological definition of schistocytes as described in the ICSH recommendations before performing microscopic assessment were as follows. Firstly, in the past, there was no standardized morphologic criteria for schistocytes. Consequently, reporting of schistocytes was subjective and variable among observers, as described by Lesesve JF.^5^ Even though a guideline for the identification and quantification of schistocytes was recently introduced^5^, it has not yet been implemented at our centre. Secondly, the three observers had different levels of experience. Based on years of experience determining schistocytes microscopically, the haematologist had the most experience, followed by the technician and the haematology resident. Therefore, we wanted to make sure that all observers had the same standardized knowledge for determining schistocytes.

The reason for selecting three observers was to see if we could identify one representative observer to examine schistocyte count whose analysis could then be compared with that of the analyser. For example, if only two observers were chosen and the ICC between them was low, we could not identify which observer determines schistocytes better. When a third observer is included to determine a couple with a high level of agreement, any of them can examine schistocyte count for the remaining samples.

***Statistical analysis***

***Interpretation of the area under the curve (AUC) of a receiver operating characteristic curve^31^***

The AUC is used to evaluate the ability of a test to distinguish between positive and negative outcomes. It is interpreted, as follows: a test is perfect if the AUC is 1; a test is excellent if the AUC is 0.9-0.99; a test is good if the AUC is 0.8-0.89; a test is fair if the AUC is 0.7-0.79; a test is poor if the AUC is 0.51-0.69; and, a test is useless if the AUC is 0.5.

***Interpretation of Spearman’s rank correlation coefficient (r_s_)^33^***

Spearman’s rank correlation coefficient (r_s_) and its 95% confidence interval (CI) are typically interpreted, as follows: the correlation is very strong if r_s_ is > 0.8 or < -0.8; the correlation is moderately strong if r_s_ is 0.6-0.8 or -0.8-(-0.6); the correlation is fair if r_s_ is 0.3-0.5 or -0.5-(-0.3); and, the correlation is poor if r_s_ is < 0.3 or > -0.3. A positive or negative r_s_ shows the direction of the correlation.

1. **Supplementary Figure**


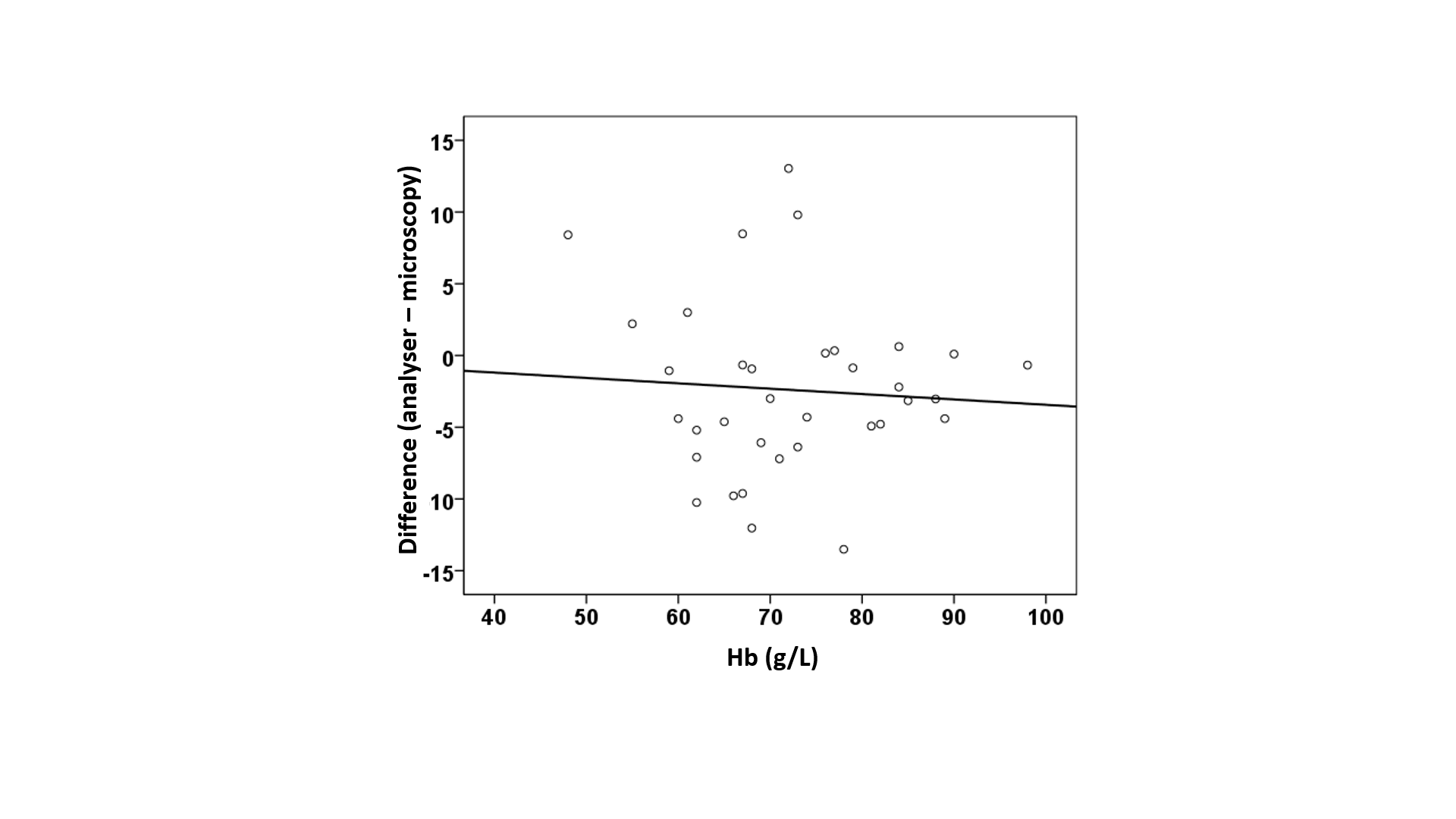


**Figure 1S. Relationship between disagreement in schistocyte percentage between the analyser and microscopy, and haemoglobin (Hb) for the thalassaemia group.** Disagreement in schistocyte measurement poorly correlates with Hb (r_s_ = 0.051, 95% CI: -0.287-0.378, P = 0.77).
